# Chronic Supplementation with a Mix of *Salvia officinalis* and *Salvia lavandulaefolia* Improves Morris Water Maze Learning in Normal Adult C57Bl/6J Mice

**DOI:** 10.3390/nu12061777

**Published:** 2020-06-15

**Authors:** Anne-Laure Dinel, Céline Lucas, Damien Guillemet, Sophie Layé, Véronique Pallet, Corinne Joffre

**Affiliations:** 1 Université de Bordeaux, INRAE, Bordeaux INP, NutriNeuro laboratory, 146 rue Léo Saignat, 33076 Bordeaux, France; sophie.laye@inrae.fr (S.L.); Veronique.Pallet@enscbp.fr (V.P.); corinne.joffre@inrae.fr (C.J.); 2 NutriBrain Research and Technology Transfer, Nutrition et Neurobiologie Intégrée, UMR 1286, 33076 Bordeaux, France; celine.lucas@nutribrain.fr; 3 Nexira, 129 Chemin de Croisset, 76000 Rouen, France; d.guillemet@nexira.com

**Keywords:** *Salvia officinalis*, *Salvia lavandulaefolia*, cognition, short-term memory, CaMKII, spatial memory, navigation strategies

## Abstract

**Background:** Two different species of sage, *Salvia officinalis* and *Salvia lavandulaefolia*, have demonstrated activities in cognitive function during preclinical and clinical studies related to impaired health situations or single administration. Different memory processes have been described to be significantly and positively impacted. **Objective:** Our objective is to explore the potential of these *Salvia*, and their additional activities, in healthy situations, and during prolonged administration, on memory and subsequent mechanisms of action related to putative effects. **Design:** This mouse study has implicated four investigational arms dedicated to control, *Salvia officinalis* aqueous extract, *Salvia lavandulaefolia*-encapsulated essential oil and a mix thereof (Cognivia™) for 2 weeks of administration. Cognitive functions have been assessed throughout Y-maze and Morris water maze models. The impact of supplementation on lipid peroxidation, oxidative stress, neurogenesis, neuronal activity, neurotrophins, neurotrophin receptors, CaM kinase II and glucocorticoid receptors has been assessed via post-interventional tissue collection. **Results:** All *Salvia* groups had a significant effect on Y-maze markers on day 1 of administration. Only the mix of two *Salvia* species demonstrated significant improvements in Morris water maze markers at the end of administration. Considering all biological and histological markers, we did not observe any significant effect of *S. officinalis*, *S. lavandulaefolia* and a mix of Salvia supplementation on lipid peroxidation, oxidative stress and neuronal plasticity (neurogenesis, neuronal activity, neurotrophins). Interestingly, CaM kinase II protein expression is significantly increased in animals supplemented with Salvia. **Conclusion:** The activities of *Salvia* alone after one intake have been confirmed; however, a particular combination of different types of Salvia have been shown to improve memory and present specific synergistic effects after chronic administration in healthy mice.

## 1. Introduction

*Salvia* (sage) is an aromatic plant from the Lamiaceae family that can be found worldwide. In total, 900 species of *Salvia* are distributed throughout the world, but common species include *Salvia* (*S.) officinalis* (common sage), *S. miltiorrhiza* (Chinese sage), *S. lavandulaefolia* (Spanish sage), *S. fruticosa* (Greek sage), *S. sclarea* (clary sage) and *S. hispanica* (chia). It is one of the most popular herbals used in traditional medicines, notably in Mediterranean culture. From Ancient Greece and Rome to the present day, *Salvia* species have been traditionally used for the treatment of a range of problems including digestive and circulation disturbances, bronchitis, coughs, asthma, angina, mouth and throat inflammation, depression and excessive sweating [1]. The most familiar, *S. officinalis*, comes from the Latin word meaning ‘to heal’ and is widely used in both culinary and medicinal preparations.

However, *Salvia officinalis* and *Salvia lavandulaefolia* species have also been specifically proposed to enhance “head and brain” function, improve normal cognition or protect against aged-related cognitive decline [2,3,4,5]. 

The prevention of cognitive alteration, especially at different critical periods of life like perinatality and aging, is a prominent challenge for medicine and appears as a major public health issue. Furthermore, in the context of a modern lifestyle characterized by an increasing overstimulation by a tremendous diversity of stimuli for cognitive functions through media and electronic sources of data, attention and memory are continuously requested. Higher capacities in focusing on high value information, reasoning and recording are probably a contemporary challenge even for healthy, active people. The nootropics properties of both *Salvia* species started to be described with preclinical and clinical evidence [6]. At an in vivo preclinical level, six studies have demonstrated the positive impact of extracts of *S. officinalis* on memory retention through different proposed mechanisms of action such as the inactivation of acetyl-cholinesterase, different antioxidant activities and the beta-adrenergic pathway [7,8,9,10,11,12]. However, all of those studies have investigated the effects of *S. officinalis* using significantly impaired cognition models, either with acute administration or non-oral administration (intraperitoneal). To our knowledge, *S. lavandulaefolia* has only had the benefit of in vitro trials, where terpenoids form essential oils have exhibited antioxidant activities and the inactivation of acetyl-cholinesterase [13,14,15]. In healthy subjects, clinical studies with *S. officinalis* or *S. lavandulaefolia* have demonstrated a beneficial effect on cognition after a single oral intake. More precisely, in three clinical studies enrolling young subjects, with an acute and dose-dependent design against a placebo, *S. lavandulaefolia* essential oil significantly improves immediate word recall [3], delayed word recall [16] and the “speed of memory” factor [17]. In the same manner, the single intake of *S. officinalis* leaves demonstrated a significant improvement in the Stroop Color Word test in a young population [16]. The acute effects on the cognitive performance of an extract of *S. officinalis* leaves in elder adults were also investigated. The authors reported an effect of sage supplementation on secondary memory performance (word recognition, picture recognition, immediate word recall and delayed word recall) [18]. Interestingly, in Alzheimer’s patients, 16 weeks of administration of *S. officinalis* leaf extract significantly improved cognitive function [19].

Considering the state of the art of the effect of *S. lavandulaefolia* essential oil and *S. officinalis* leaves on cognitive ability during pathologies, demonstrated in preclinical and clinical studies through acute administration or impaired situation, evaluating the impact of *Salvia* in healthy individuals and during a chronic administration could be a promising challenge for highlighting improvements in cognition in normal situations. 

The objective of this project was to evaluate the impact of acute and chronic administration of *S. Lavandulaefolia essential oil* encapsulated on acacia gum, *S. officinalis* leaf extract and a mix of both types of *Salvia* on cognitive ability in order to understand the mechanisms underlying their functional impact on memory processes in healthy adult mice. Firstly, the acute effect of *Salvia* administration was characterized via the Y-maze paradigm, then the chronic impact of *Salvia* supplementation was assessed in the Morris water maze and, finally, the neurobiological correlates underlying these effects were investigated.

## 2. Materiel and Methods

### 2.1. Animals and Nutritional Supplementation

Animal husbandry and experimental procedures were in accordance with the EU Directive 2010/63/EU for animal experiments and approved by the national ethical committee for care and use of animals (approval ID A16320). Every effort was made to minimize animal suffering and the number of animals used. Seven-week-old male C57Bl/6J mice from Janvier Labs (Le Genest-Saint-Isle, France) were housed in polypropylene cages and maintained in a temperature- and humidity-controlled pathogen-free facility with a 12 h light-dark cycle abd ad libitum access to food and water. Mice were handled daily for 1 week before experiment onset to minimize stress reactions due to manipulation. Mice were fed with A04 diet (Safe, Augy, France). They were supplemented by gavage (feeding probe V0105040, ECIMED, Boissy-Saint-Leger, France) with *S. lavandulaefolia* essential oil encapsulated with acacia gum (83.3 mg/kg/day), or *S. officinalis* aqueous leaf extract (166.7 mg/kg/day) or a mix of both vegetal extracts (Cognivia™; 250 mg/kg/day) or water for the control group, each morning for two weeks. *S. lavandulaefolia* essential oil was characterized for its content in terpenoids (as eucalyptol, camphor, α-, β-pinene and others) and *S. officinalis* aqueous leaf extract was characterized for its content in polyphenols (as rosmarinic acid, apigenin glucosides, luteolin glucosides and others). The encapsulation of essential oil was performed using acacia gum in solution with a specific shear stress in order to create dispersed and stabilized small oil droplets of essential oil (1 to 5 µm). Finally, the combination of both extracts was done using exactly the same materials. After the first day of gavage, mice were subjected to a Y-maze test to evaluate the impact of an acute dose on long-term memory. After two weeks of gavage, mice were submitted to a Morris water maze to evaluate the impact of a chronic dose on learning and long-term memory. At the end of the behavioral protocol, mice were sacrificed, and the brain structures implicated in memory (hippocampus, prefrontal cortex) and red blood cells were collected and frozen at −80 °C.

### 2.2. Behavioral Testing

#### 2.2.1. Y-Maze 

Spontaneous spatial recognition in the Y-maze was used as a hippocampal-dependent test, as previously described [20]. The apparatus consisted of a Y-shaped acrylic maze with three identical arms (34 × 8 × 14 cm). The floor was covered with corncob litter and was mixed between each trial in order to remove olfactory traces. Visual cues were placed on the walls of the testing room and kept constant during the whole test. The discrimination of novelty versus familiarity was based on the various environmental cues that the mouse can perceive from each arm of the Y-maze. In the first trial of the test (acquisition), one arm was closed with a door and mice were allowed to freely visit the two other arms for 5 min. After a 30-min intertrial interval (ITI), mice were again placed in the ‘start’ arm for the second trial (retrieval) and allowed free access to all three arms for 5 min. ‘Start’ and closed arms were randomly assigned to each mouse. Arm entries were defined as all four paws entering the arm. The animals were video tracked (SMART system; Bioseb, Vitrolles, France) to analyze the time spent in the different arms. Analyses were based on the time spent exploring the novel and familiar arms during 5 min of the second trial. 

#### 2.2.2. Spatial Learning and Reference Memory in the Morris Water Maze 

Chronic gavage was realized in animals after 14 days and learning and spatial memory were evaluated using a Morris water maze [21]. 

##### Training Phase 

Two weeks after the beginning of the diets, spatial learning and memory were assessed in a Morris water maze (150 cm in diameter, 50 cm-high) filled with white water (22 °C) and surrounded with distal extramaze cues. Before being trained, animals were handled for 1 min a day for 2 days. Mice were then familiarized with water and swimming during two familiarization days (day 1 and day 2) where they had to find a visible platform in the center of a small pool (60 cm diameter) surrounded with curtains (three consecutive trials a day; 60 s-cut-off). On day 0, to evaluate visuomotor deficits, mice were given six trials (90 s-cut-off) to find a visible platform pointed out with a cue in the Morris water maze that was surrounded with white curtains. During the training sessions (days 1–4), animals were required to locate the submerged platform by using distal extramaze cues. They were trained for six trials a day (90 s-cut-off) with an intertrial interval of 5 min for four consecutive days. In order to facilitate spatial learning, mice were introduced from four different starting points, in a randomized daily order. The speed, the latency and the distance to reach the platform as well as the swim path of each trial were recorded by an Imetronics videotracking system (France). The daily swim path efficiency was calculated as the ratio of the shortest possible length to the effective swim path length. 

##### Probe Test 

Seventy-two hours after the last training session, the platform was removed from the pool and spatial memory was evaluated for 60 s. The percentage of distance traveled in the four quadrants was recorded using the SMART system (San Diego Instruments, San Diego, CA, USA). The quadrant where the platform was located during training is referred to as the target quadrant. Additionally, the number of annulus crossings and the mean proximity to the platform were assessed during this test as reliable measures of probe test performance [22]. 

##### Analyses of Navigation Strategies

For the probe test, the navigation path was analyzed with the videotracking system (Imetronics, Pessac, France) and assigned to one of the eleven strategies. The categorization scheme was adapted from those developed previously [23,24,25,26,27]. These strategies were divided into two main categories: non-spatial vs. spatial strategies. Non-spatial strategies included first ‘‘global search’’ strategies: ‘‘peripheral looping’’ (persistent swimming around the outer 15 cm of the pool, including thigmotaxis), ‘‘random’’ (searching the entire tank, >75% surface coverage), ‘‘circling’’ (swimming in tight circles, possibly with some net directional movement), and then ‘‘local search’’ strategies: ‘‘scanning’’ (searching restricted to a limited, often central, portion of the tank, >15% and <75% of surface coverage), ‘‘chaining’’ (circular swimming at an approximately fixed distance greater than 15 cm from the wall), ‘‘repeated incorrect’’ (swimming in a precise direction that does not contain the platform and repeat the same trajectory several times), and ‘‘focal incorrect’’ (searching intently a small portion of the tank that does not contain the platform). Spatial strategies included ‘‘repeated correct’’ (swimming in direction of the platform and repeat the same trajectory several times), ‘‘focal correct’’ (swimming and searching intently in the zone containing the platform), ‘‘spatial indirect’’ (swimming indirectly to the platform with eventually 1–2 loops) and ‘‘spatial direct’’ (swimming directly to the platform). 

### 2.3. Biochemical/Histological Measurements

#### 2.3.1. Evaluation of Lipid Peroxidation (MDA Dosage) in Cortex

The quantification of lipid peroxidation is essential to assess oxidative stress in pathophysiological processes. Measuring the end products of lipid peroxidation is one of the most widely accepted assays for oxidative damage. The end products of lipid peroxidation are reactive aldehydes such as malondialdehyde (MDA) as natural byproducts. A Lipid Peroxidation Assay Kit (ab118970, Abcam, Paris, France) was used to detect MDA. MDA in the sample reactions with Thiobarbituric Acid (TBA) to generate a MDA–TBA adduct, which can easily be quantified colorimetrically (OD 532 nm). MDA content was expressed in nmoles/mg protein. Proteins were measured colorimetrically (OD 562 nm) using a BCA (bibinchoninic acid assay) kit (Uptima, Interchim, Montluçon, France).

#### 2.3.2. Measurement of Superoxide Dismutase Activity in Cortex in Red Blood Cells

Superoxide dismutase (SOD) is one of the most important anti-oxidative enzymes. It catalyzes the dismutation of the superoxide anion into hydrogen peroxide and molecular oxygen. Superoxide Dismutase Activity Assay (ab65354, Abcam, France) is a sensitive kit using WST-1 that produces a water-soluble formazan dye upon reduction with superoxide anion. The rate of the reduction with a superoxide anion is linearly related to the xanthine oxidase (XO) activity, and is inhibited by SOD. Therefore, the inhibition activity of SOD can be determined by a colorimetric method (OD 450 nm). 

SOD activity (inhibition rate %) was calculated with the following formula:

= ((Ablank1 − Ablank3) − (Asample − Ablank2)) × 100/(Ablank1 − Ablank3)

A: absorbance; blank 1: H_2_O; blank 2: sample; blank 3: H_2_O.

#### 2.3.3. Immunohistochemical Detection of Doublecortin (DCX)-Positive Cells 

Neurogenesis in the hippocampus was evaluated by determining the number of immature neurons in the dentate gyrus characterized by the endogenous marker doublecortin (DCX) (Santa Cruz Biotechnology, Santa Cruz, CA, USA), a cytoplasmic protein expressed transiently in newborn neurons only [20,28]. Neurogenesis is largely implicated in cognitive processes. The intensity of DCX-labeling was first measured in the dentate gyrus of mice, as an index of neuronal proliferation. After transcardiac perfusion with phosphate buffered saline (PBS, pH 7.4), followed by 4% paraformaldehyde (PFA), brains were removed, postfixed for 2 h in PFA, cryoprotected in 30% sucrose for 24 h, snap frozen in liquid nitrogen, and stored at −80 °C before sectioning. Free-floating coronal sections (40 μm) containing the hippocampus were collected on a cryostat for immunohistochemistry. The results were expressed as the number of total and immature neurons/mm^3^.

#### 2.3.4. Immunohistochemical Detection of c-Fos Positive Cells 

C-Fos imaging was used as a neuronal activity reporter to map neurons activated in the hippocampus after the memory test. To analyze cellular activation in the hippocampi of mice under study, we measured the expression of c-Fos by immunohistochemistry. 

After transcardiac perfusion with PBS (pH 7.4), followed by 4% paraformaldehyde, brains were removed, post-fixed overnight, cryoprotected in 30% sucrose for 24 h, snap frozen in liquid nitrogen, and stored at −80 °C before sectioning. Free-floating 40 µm coronal sections through the hippocampus (from −0.9 mm to −3.1 mm relative to bregma) were collected on a cryostat for immunohistochemistry. 

To analyze cellular activation in the hippocampus, we measured the expression of c-Fos by immunohistochemistry as previously described (Labrousse et al., 2012). Briefly, sections were incubated overnight at 4 °C in rabbit polyclonal antiserum raised against c-Fos (reference: J1011, Santa Cruz Biotechnology, Santa Cruz, CA, USA), diluted 1:1000 in PBS containing 0.3% Triton X-100, 3% bovine serum albumin, before being incubated for 2 h with biotinylated goat antirabbit antibody (1:2000; Vector laboratories, Burlingame, CA, USA). Sections were then incubated for 2 h with avidin-biotin peroxidase complex (1:1000; Vectastain ABC kit, Vector laboratories, Burlingame, CA, USA), and labeling was revealed with diaminobenzidine using the nickel-enhanced glucose oxidase method. Sections were then mounted onto gelatin-coated glass slides. Negative controls in which the primary antibody was omitted did not show any immunolabeling. Cells were visualized using 20x magnification (Olympus BX51) and quantification was carried out using ImageJ software (http://imagej.nih.gov.gate2.inist.fr/ij/) as previously described [29] in grids randomly placed in the dorsal hippocampus in both hemispheres (size, 150 × 150 µm and spacing, 100 × 100 µm). Results were expressed as the number of activated neurons/mm^2^.

#### 2.3.5. Measurement of Neurotrophins, Neurotrophin Receptor, CaM Kinase II and Glucocorticoid Receptors by Western Blot

Western blot analyses of the brain-derived neurotrophic factor (BDNF) and its tyrosine receptor kinase B (TrkB), the glucocorticoid receptor (GR) and its phosphorylated form (P-GR), as well as calmodulin-dependant protein kinase II (CaMKII), were performed according to a previously published method [20,30]. Briefly, the hippocampus and prefrontal cortex were homogenized with a Precellys 24 system (Bertin Technologies, Aix en Provence, France) in lysis buffer. An equal amount of proteins was loaded onto sodium dodecylsulfate – polyacrylamide gels (SDS-PAGE) (10% acrylamide) and transferred onto polyvinylidene difluoride (PVDF) membranes (Millipore, Billerico, MA, USA). Membranes were incubated overnight at 4 °C with the following primary antibodies: anti-BDNF (1:1000; Abcam, Paris, France), anti-TrkB (80E3, 1:1000; Cell Signaling Technology, Boston, MA, USA) anti-glucocorticoid receptor (GR) (1:1000; Santa Cruz Biotechnology, Santa Cruz, CA, USA), anti-phospho-GR (Ser211) (1:5000; Cell Signaling Technology, Boston, MA, USA), anti-CaMKII (1:1000; Cell Signaling Technology, Boston, MA, USA) and anti-Actin (1:2500; Sigma, St-Louis, MO, USA). After being washed, membranes were incubated with peroxidase-conjugated secondary anti- rabbit antibody for 1 h (1:5000; Jackson ImmunoResearch laboratories, Westgrove, PA, USA). Staining was revealed with the ECL-Plus Western blotting detection system (Perkin Elmer, Forest City, CA, USA) or Lumina Forte Western horseradish peroxidase (HRP) substrate (Millipore, Billerico, MA, USA). Chemiluminescence was captured by a Chemidoc detection system and quantified by Image Lab software (Biorad, Hercules, CA, USA).

### 2.4. Statistical Analysis

All data are expressed as means ± SEM. The swim velocity and distance to reach the platform in the Morris water maze were compared by a two-way ANOVA test. Data obtained for time spent in the Y-maze and distance and the number of entries in the Morris water maze were analyzed using a two-way ANOVA test with diet and arm/quadrant as between factors, followed by Fischer’s Least Significant Difference (LSD) post-hoc comparisons when appropriate. Biochemical data were compared by a one-way ANOVA test, followed by a Dunnett post-hoc when appropriate. A *p* value < 0.05 was considered as significant.

## 3. Results

### 3.1. Acute Salvia Supplementation Improved Mice Cognitive Ability in Y-maze Test

**Figure 1:**
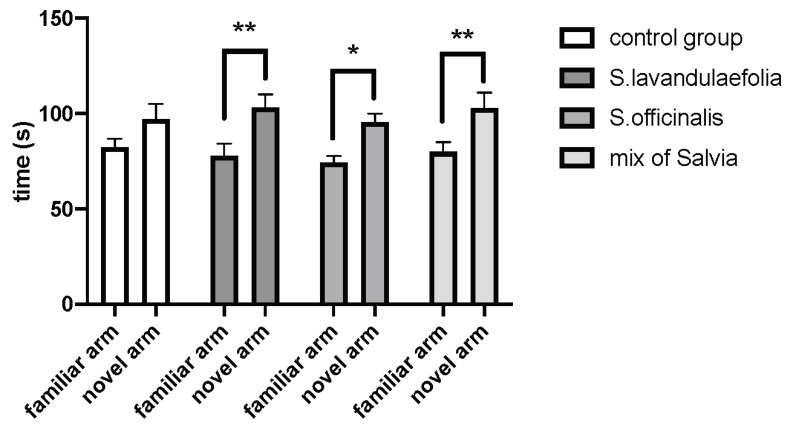
Acute Salvia supplementation restores short-term memory. Spatial memory performance was evaluated in the Y-maze test by measuring time spent exploring the novel and the familiar arms after a 1-h retention intertrial interval (ITI). * *p* < 0.05, ** *p* < 0.01, for the familiar arm compared to the novel arm. n = 9–10/group.

The Y-maze test was performed 1h after the first gavage (day 1). In this test, the time spent to explore the novel arm was compared to the time spent to explore the familiar arm during the restitution phase (Figure 1). The two-way ANOVA revealed the effect of the arm (F(1.69) = 22, *p* < 0.0001). In control mice, the amount of time spent in the novel arm was not significantly different to the amount of time spent in the familiar arm, indicating that control mice did not distinguish the novel arm. Interestingly, animals from groups containing *Salvia* spent significantly more time in the novel arm than in the familiar arm (group *S. lavandulaefolia*, t = 2.963, df = 69, *p* = 0.0042; group *S. officinalis*, t = 2.248, df = 69, *p* = 0.0278; Mix group, t = 2.672, df = 69, *p* = 0.0094), indicating that animals classified the novel arm through a hippocampal process.

### 3.2. Chronic mix of Salvia Supplementation Improved Cognitive Ability in Morris Water Maze

**Figure 2:**
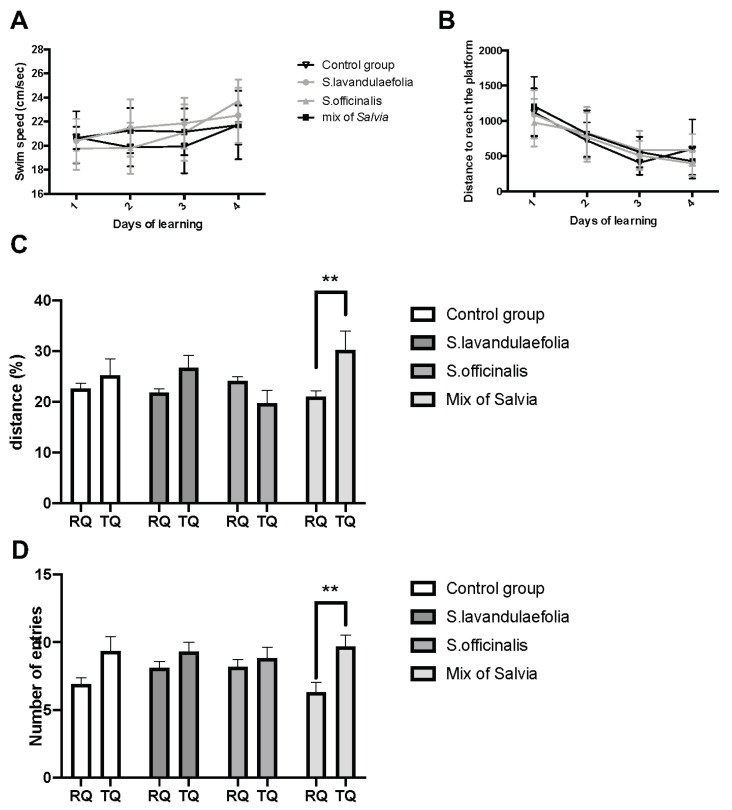
Chronic mix of Salvia supplementation improved long-term memory. Long-term memory performance was evaluated in the Morris water maze. (**A**) Swim speed during learning (day effect: ** *p* < 0.01 by 2-way ANOVA; n = 10 per group). (**B**) Distance covered to reach the platform over the 4 consecutive days of spatial learning (day effect *p* < 0.0001 by 2-way ANOVA with repeated measures). (**C**) Percentage of distance travelled in quadrants (remaining quadrants vs. target quadrant) during the probe test. (** *p* < 0.01, 2-way ANOVA, n=10 per group). (**D**) Number of entries in remaining quadrants compared to target quadrant (** *p* < 0.01, 2-way ANOVA, n = 10 per group).

Swim velocity was evaluated in a Morris water maze test to eliminate all bias due to visual and motor deficits. We did not observe any effect of the supplementation on swim speed during learning, but we did observe a day effect (time effect, F(3.108) = 3.435, *p* < 0.0001) (Figure 2A).

During spatial learning (4 days, six trials per day and per animal), all groups learned to reach the submerged platform (time effect, F (3.108) = 40.38, *p* < 0.0001) (Figure 2B). There was no significant effect of the supplementation.

During the restitution test, the distance traveled in each quadrant and the entry numbers in each quadrant were analyzed. A two-way ANOVA revealed a significant interaction effect (diet x quadrant) (F(3.70) = 3.1, *p* = 0.0296).

Animals supplemented with the mix of *Salvia* traveled significantly more distance in the target quadrant (TQ) compared to the remaining quadrants (RQ) (*p* = 0.0051) (Figure 2C).

The number of entries in the target quadrant compared to the remaining quadrants was also increased in the mix group (quadrant effect, F(1.70) = 13, *p* = 0.0004, *p* = 0.0014) (Figure 2D).

Our results showed that supplementation with the mix of both types of *Salvia*, but not with *Salvia* alone, improved cognitive ability in mice during the restitution phase of the Morris water maze test. This difference was not associated with a improvment of learning ability.

### 3.3. Chronic Mix of Salvia Supplementation Modulated Strategies during Spatial Memory

**Figure 3:**
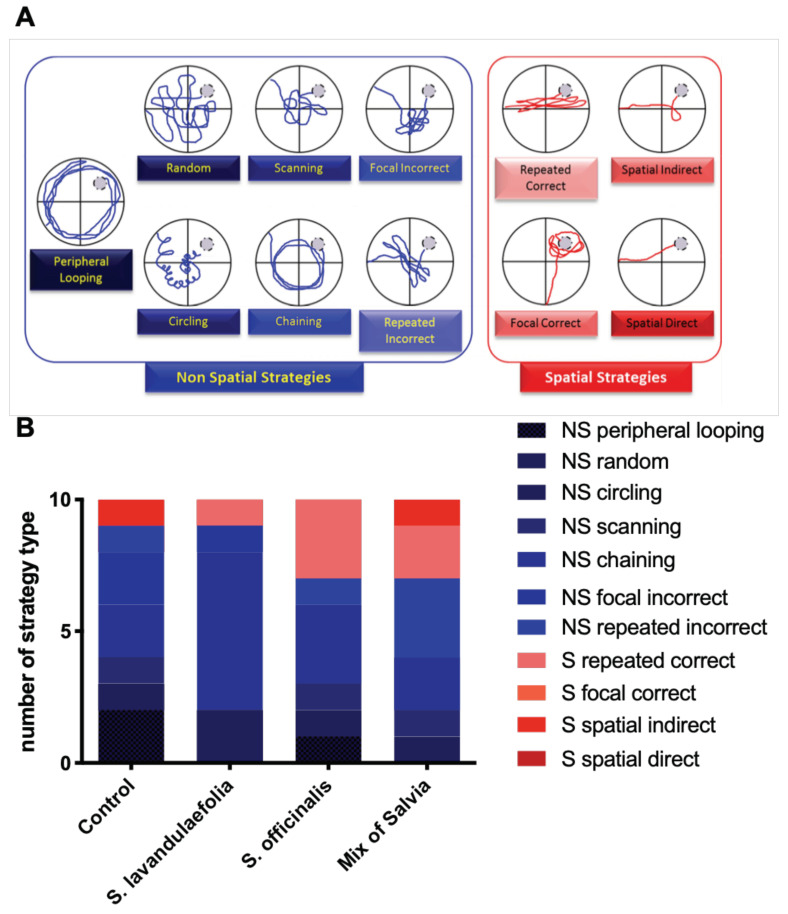
Chronic mix of Salvia supplementation modulated navigation strategies during probe test. (**A**) Representative path patterns with non-spatial (blue) and spatial (red) strategies used to reach the hidden platform (from Bensalem et al., 2016). (**B**) Number of type of strategies used by group during probe test to reach the platform location.

To characterize the impact of Salvia supplementation on spatial memory, the navigation path of the probe test was qualitatively analyzed (Figure 3A,B). A detailed analysis of the navigation path revealed that control mice used an essentially non spatial strategy with a lot of peripheral looping pathways. The strategies ‘‘focal incorrect’’ and ‘‘repeated incorrect’’, categorized as non-spatial strategies, are, in fact, intermediate, suggesting that mice may use distal cues in an erroneous manner and that their cognitive map is not fully acquired.

Interestingly, animals supplemented with *S. officinalis* and a mix of *Salvia* adopt a 30% of spatial strategy, whereas control and *S. lavandulaefolia* mice adopt only 10% of spatial strategy. In particular, animals supplemented with a mix of *Salvia* developed two types of spatial strategies to reach the target quadrant (repeated correct and spatial indirect).

### 3.4. Chronic Mix of Salvia Supplementations Did Not Impact Oxidative Stress

**Figure 4:**
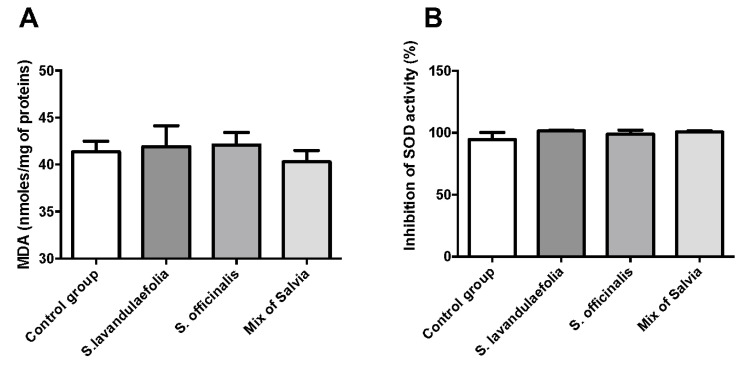
Chronic mix of Salvia supplementation did not impact oxidative stress. (**A**) Evaluation of lipid peroxidation by malondialdehyde (MDA) dosage (n = 6 per group). (**B**) Evaluation of the inhibitory activity of superoxide dismutase (SOD) (% of inhibition) (n = 6 per group).

To investigate the effect of Salvia supplementation on oxidative stress, we evaluated the impact of *Salvia* supplementation on lipid peroxidation and superoxide dismutase activity. Lipid peroxidation was evaluated by MDA dosage in the cortex (Figure 4A). We did not observe any significant difference in MDA concentration between groups. We evaluated the inhibitory activity of SOD (% of inhibition) in red blood cells and in the prefrontal cortex of mice. The results obtained did not reveal any significant difference in the inhibitory activity in red blood cells (data not shown) and the prefrontal cortex (Figure 4B).

### 3.5. Chronic Mix of Salvia Supplemention Did Not Impact Hippocampic Neurogenesis

**Figure 5:**
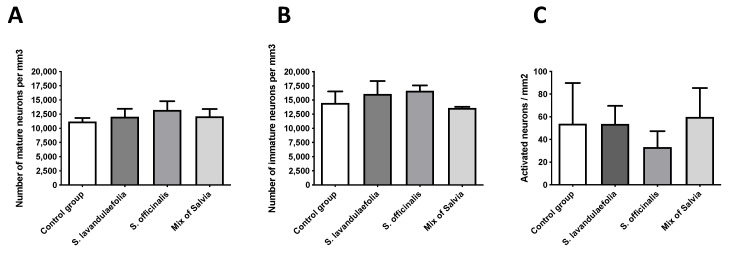
Chronic mix of Salvia supplementation did not impact neurogenesis and neuronal activity. (**A**) Number of total neurons by mm^3^ evaluated with doublecortin (DCX) staining in dentate gyrus of hippocampus (n = 4 per group). (**B**) Number of immature neurons by mm^3^ evaluated with doublecortin (DCX) staining in dentate gyrus of hippocampus (n = 4 per group). (**C**) Evaluation of neuronal activity with c-Fos staining (n = 4 per group). All analyses were done using a one-way ANOVA.

To measure the impact of *Salvia* on neurogenesis, we evaluated the proliferation of doublecortin (DCX)-positive cells in the dentate gyrus of the hippocampus. New neuron numbers were expressed in the number of neuron/mm^3^ of structure for each group. We did not show any significant difference in total neuron numbers (Figure 5A) or in immature neuron numbers (Figure 5B).

### 3.6. Chronic Mix of Salvia Supplementation Did Not Impact Neuronal Activity

c-Fos immunochemistry was used to evaluate the impact of *Salvia* on neuronal activity in ventral hippocampus. Neuronal activity was expressed in neurons’ number/mm^2^ of structure for each group. We did not observe any significant difference in neuronal activity between each group (Figure 5C).

### 3.7. Chronic Mix of Salvia Supplementation Did Not Impact Neurotrophic Factor But Increased CaMKII Protein Expression

**Figure 6:**
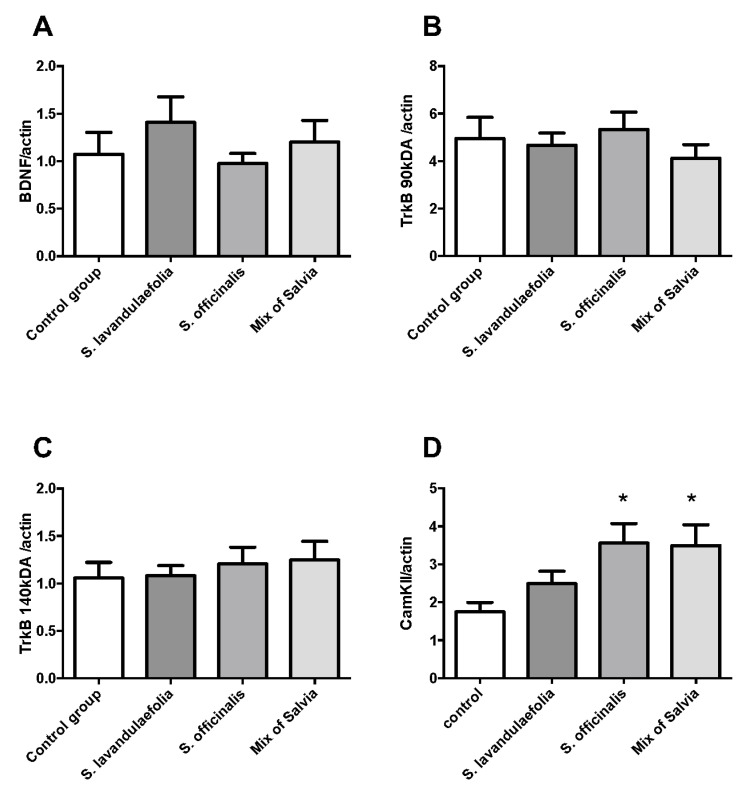
Chronic mix of Salvia supplementation did not impact neurotrophic factor but increase CaMKII protein expression. The protein expression of BDNF (**A**), TrkB (90kDa) (**B**), TrkB (140kDa) (**C**) and CamKII (**D**) compared to actin was evaluated by Western blot. All analyses were done using a one-way ANOVA (* *p* < 0.05 n = 6 per group).

We measured the production of the neurotrophic factor BDNF and its receptors (TrkB high, TrkB low) by Western blot in the prefrontal cortex of mice. Neither BDNF (Figure 6A) nor TrkB expression (Figure 6B,C) was impacted by *Salvia* supplementation. CaMKII protein expression is significantly increased (F(3.19) = 3.587, *p* = 0.033) in *S. officinalis* fed animals and mix-fed animals compared to control animals (t = 2.793, Df = 19, *p* = 0.0297 and t = 2.689, Df = 19, *p* = 0.0368 respectively) (Figure 6D).

### 3.8. Chronic Mix of Salvia Supplementation Did Not Impact the Expression of Glucocorticoid Receptor and Its Phosphorylated Form in the Prefrontal Cortex of Mice

**Figure 7:**
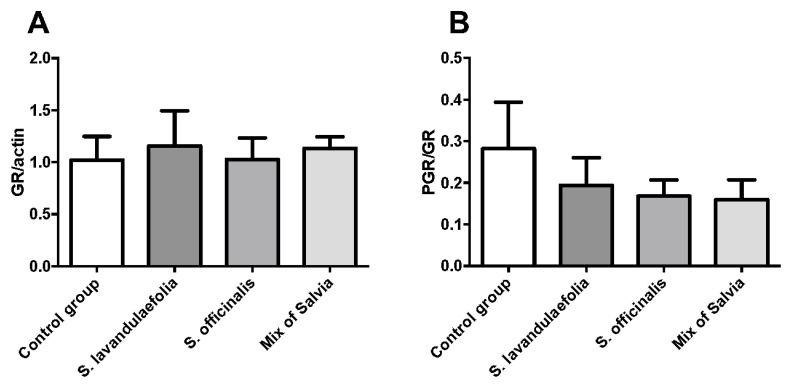
Chronic mix of Salvia supplementation did not impact glucocorticoid receptor (GR) and its phosphorylated form (P-GR) protein expression. The protein expression of GR (**A**) and P-GR (**B**) compared to actin was evaluated by Western blot (n = 6 per group).

Interestingly, cognitive ability and performance can be impacted by stress and hypothalamic-pituitary-adrenal (HPA) axis activity (Sandi C. et al., 2013). We investigated the impact of *Salvia* supplementation on HPA axis activity through the measurement of the expression of the glucocorticoid receptor and its phosphorylated form in the prefrontal cortex. We did not observe any modulation in the glucocorticoid receptor protein level (Figure 7A) or the phosphorylated glucocorticoid receptor/glucocorticoid receptor ratio (Figure 7B).

## 4. Discussion

In the present study, we have shown, for the first time, that *S. lavandulaefolia* and *S. officinalis* improved the memory of healthy adult mice and presented a synergistic effect. Indeed, we evidenced that both an acute and chronic mix of *Salvia* supplementation enhanced the spatial hippocampus-dependent memory.

We first demonstrated that an acute intake of *S. lavandulaefolia, S. officinalis* and a mix of both *Salvia* species improved spatial working memory in healthy adult mice in a Y-maze test, whereas control mice presented an altered cognitive ability. These conclusions are in accordance with the literature, which has demonstrated an acute effect on cognitive functions related to memory after a single administration in rodents and humans for both types of *Salvia* products [7,17]. Here, we should also note that the encapsulation of essential oil by acacia gum (for protection against oxidation and evaporation processes) did not notably affect the kinetics of its physiological activity, as demonstrated via the memory improvement after a single administration. This alteration of cognitive ability in the control group could be induced by the force-feeding protocol. The restraint procedure associated with gavage induces a stress in animals [31]. However, oral gavage presents an advantage over other methods of oral administration to be more accurate and reliable for administering substances into the gastro-intestinal tract, as it eliminates risks of variability in intake between individual animals. It is well described that the exposure to high stress impairs the formation of explicit memories and, more generally, of hippocampus-dependent memory [32]. As *Salvia*-supplemented mice did not present this alteration, we hypothesized that they react better to stress. To confirm these results, a novel Y-maze was realized at the end of the oral feeding procedure, and the same results were obtained, confirming that alterations in the normal group were probably due to the anxiety mediated by gavage. To investigate this new and interesting hypothesis, novel experimentation should be done to evaluate corticosterone secretion and the expression of stress-responsive genes.

We also demonstrated a synergistic effect of the 14-day chronic supplementation of both species of *Salvia* on cognitive ability characterized by an increase in the distance travelled and of the number of entries in the target quadrant. The improvement of cognitive ability could not be explained by a difference in learning ability, since spatial learning was not affected by the supplementation. Indeed, all groups learned to find the immerged platform with a similar efficiency.

The hippocampus is one of the brain structures involved in spatial learning. Long-term memories are associated with molecular changes [33,34,35], such as the synthesis of new mRNAs and proteins [36,37,38]. Various signaling pathways involved in the control of de novo protein synthesis converge in the expression of many genes associated with synaptic plasticity underlying spatial learning and memory [39,40,41,42,43,44,45]. Among them is neurogenesis in the subgranular zone of the hippocampal dentate gyrus [46,47,48], which is highly implicated in spatial learning memory [49]. Within the hippocampal region, the dentate gyrus continues to produce granule neurons throughout adulthood [50,51,52]. An increasing number of reports suggest that adult hippocampal neurogenesis is involved in hippocampal-mediated learning. Indeed, the hippocampus is implicated in various forms of memory and, notably, in spatial memory, and it has been shown that conditions that increase memory performance also enhance neurogenesis [53,54,55,56].

Moreover, it has also been demonstrated that c-Fos expression in the hippocampus may be obligatory for spatial memory formation [57]. To understand the mechanisms involved in *Salvia*-induced memory enhancement, neurogenesis and c-Fos activation were evaluated. In our model, the number of total and immature neurons was not modulated by the supplementation. This absence of neurogenesis modulation could be explained by an adaptive strategy of mice to realize the task and perform in the Morris water maze. Two types of frames are used to represent spatial information: the egocentric frame, which includes spatial information about the location of the individual in the environment, and the allocentric frame, which involves spatial information about the position of objects relative to each other [58]. To manage successful navigation and provide healthy and efficient spatial memory, both frames must be combined and different spatial strategies, depending on the environmental cues, are required [59]. In the Morris water maze test, animals were introduced at multiple starting points and this procedure theoretically promotes the formation of an allocentric cognitive map [60]. The allocentric frame is also associated with the formation of new neurons since it has been shown that the absence of new hippocampal neurons selectively affects the spatial memory performance associated to allocentric, but not egocentric, strategy [24,61]. In our model, we hypothesize that animals supplemented with a mix of *Salvia* could not promote an allocentric cognitive map to find the hidden goal. Moreover, the Morris water maze is considered as a test of spatial learning and memory [21,62], but this task can also be solved by alternative non-spatial strategies. We demonstrated, in this article, that diet could impact navigation patterns and that a mix of *Salvia* supplementation seemed to promote the acquisition of spatial strategies to the detriment of non-spatial ones.

In the same way, the number of activated neurons (evaluated by c-Fos positive neurons) was not increased by *Salvia* supplementation. Following spatial learning, the upregulation of immediate early genes has been extensively described. Among these genes, c-Fos is a transcription factor whose upregulation correlates with spatial learning and memory in rodents [63,64,65]. Nevertheless, it has been shown that c-Fos mutant mice exhibit normal spatial memory in the Morris water maze, suggesting that the hippocampal expression of c-Fos was not necessary for spatial learning [66]. Moreover, Shires and Aggleton demonstrated that the expression of the immediate early genes in the hippocampus were not a sufficient predictor of spatial learning [67]. C-Fos regulates the expression of BDNF in distinct neuron populations of the hippocampus [68] and BDNF is implicated in the control of neurogenesis in the dentate gyrus of the hippocampus [69]. In agreement with those findings, our results did not show a modulation of BDNF production in the supplemented or control group. Interestingly, *S. officinalis* and a mix of *Salvia* supplementation induced an increase in CaMKII protein expression that is consistent with previous work associating the modulation of CaMKII expression with the use of spatial strategies [27]. CaMKII has been proposed to underlie memory and learning processes due to its implication in many neuronal functions—notably, neurotransmitter metabolism, neuronal signal transduction (ionic and channel activity), synaptic plasticity and long-term potentiation [70,71,72]. However, to definitively determine the role of *Salvia* in neuronal function, specific pathways are yet to be elucidated (i.e., long-term potentiation and dendritic spine formation).

## 5. Conclusions

Significant effects on spatial memory for each *salvia* extract (*S. lavandulaefolia* essential oil and *S. officinalis* leaf extract) or a combination have been demonstrated after a single administration in normal adult mice. The encapsulation of *Salvia* essential oil by acacia gum still allows for acute activity. Only the combination of both extracts (Cognivia™) highlighted a significant synergistic effect on long-term memory after a chronic administration. Mechanisms of action related to the chronic effect of *Salvia* may implicate CamKII modulation. These promising findings related to normal healthy situations must be investigated further to confirm putative human benefits in the context of the chronic administration of *Salvia*. However, this study demonstrated that a mix of both *Salvia* species is a potential food supplement strategy to improve memory in adulthood and can be an innovative tool either to avoid memory loss or to elevate normal cognition.
